# Neonatal Outcomes Following Delivery in Water: Evaluation of Safety in a District General Hospital

**DOI:** 10.7759/cureus.2208

**Published:** 2018-02-20

**Authors:** Phil J Peacock, Stanley T Zengeya, Lesley Cochrane, Maxine Sleath

**Affiliations:** 1 Emergency Department, Bristol Royal Hospital for Children; 2 Department of Paediatrics, Great Western Hospital, Swindon; 3 White Horse Birth Centre, Great Western Hospital, Swindon; 4 Maternity Services, Great Western Hospital, Swindon

**Keywords:** neonatal, waterbirth, pediatrics

## Abstract

Introduction

Giving birth in water has increased in popularity over recent years, with potential benefits in terms of maternal comfort and decreased rates of instrumental delivery. Some concerns have been raised about possible adverse neonatal outcomes, including hypothermia and respiratory distress. There is not currently, however, a clear consensus in the literature. This study sought to assess the safety of delivering in water for low-risk vaginal deliveries in a District General Hospital in the United Kingdom.

Methods

Prospectively collected hospital data was obtained for all deliveries between 1 April 2014 and 31 March 2016 at the Great Western Hospital, Swindon. The dataset was limited to full-term babies born by unassisted vaginal delivery following spontaneous labour; 3507 babies were included in the analyses. Pre-specified outcomes included neonatal unit admission, Apgar scores, and temperature after delivery.

Results

During the two-year period studied, there were 592 waterbirths and 2915 non-waterbirths. There was no significant difference in rates of neonatal unit admission between waterbirths and non-waterbirths. One-minute Apgar scores were slightly higher among those born in water (P = 0.04); this difference attenuated by five minutes of age. There was no difference in temperature after delivery between the two groups.

Conclusions

An evaluation of safety in a District General Hospital has demonstrated similar postnatal outcomes among babies born in water, compared to those born on land. Further work examining longer-term outcomes would help assess whether this persists beyond the newborn period.

## Introduction

Giving birth in water has become increasingly popular in the United Kingdom (UK), since its first recognition by the Department of Health and Royal College of Midwives in 1993 and 1994, respectively. Labouring in water has potential benefits in terms of comfort for the mother, better pain relief, and decreased need for instrumental delivery [1-3]. However, there have been concerns raised in the literature about the possibility of adverse neonatal outcomes from delivery in water, including low temperature and respiratory distress [4].

There have been case reports of respiratory distress secondary to presumed aspiration during waterbirth [5-7], as well as some evidence from case reviews of a greater level of respiratory morbidity among babies born in water [8].

Conversely, a Turkish study of over 200 waterbirths and matched controls reported no difference in neonatal unit admission or Apgar score, and no incidences of neonatal infection or death in those born in water [9]. A Swiss study found no differences in terms of Apgar score, cord pH, neonatal unit admission rates or neonatal infections between waterbirths and land births [10]. A large Italian study found no difference in rates of stillbirth or neonatal death [1].

Interpretation of the existing literature is difficult given the conflicting outcomes, different study designs and variability in practice across different healthcare systems. The latest Cochrane Review on this subject concluded that further research was needed to understand the impact of waterbirth on neonatal morbidity [4]. A UK national guideline currently encourages labouring in water for pain relief, but concludes there is insufficient evidence to either support or discourage giving birth in water [11].

The Great Western Hospitals Foundation Trust is a District General Hospital in the UK, with an annual birth rate of approximately 4500. The Trust has two birthing pool rooms within its midwife-led birthing centre, a single pool room on the main delivery suite, and portable birthing pools for home births. This project sought to evaluate the safety of giving birth in water, in terms of neonatal outcomes, when compared to delivery on land.

## Materials and methods

**Figure 1:**
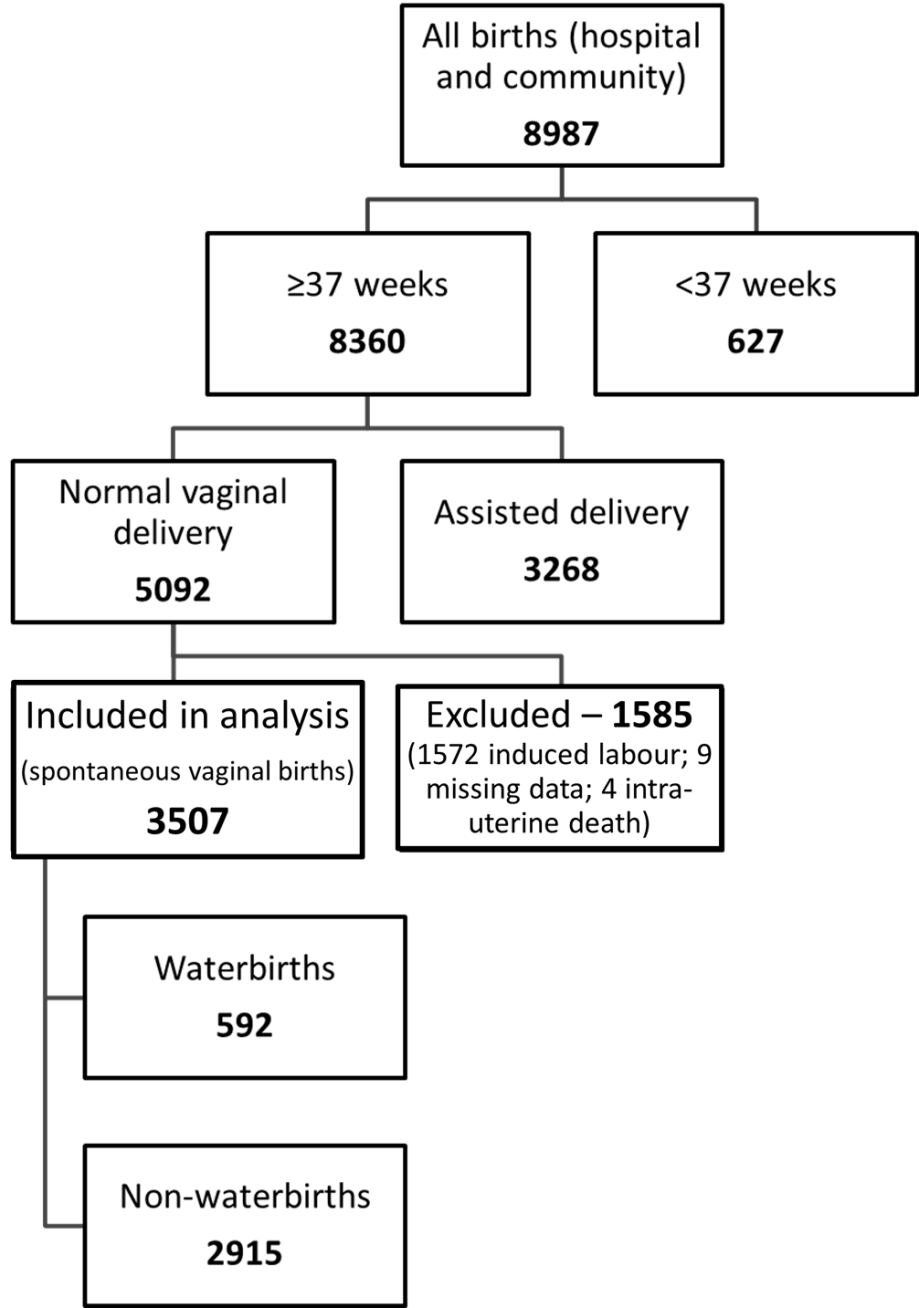
Flowchart showing selection of sample for analysis.

Details of all deliveries in the period 1 April 2014-31 March 2016 were obtained from prospectively collected routine hospital data and anonymised in line with Information Governance guidelines. All unassisted vaginal deliveries at full-term during the period were identified (n = 5092). Deliveries where labour was induced (n = 1572) or insufficient details on labour recorded (n = 9) were excluded, leaving a dataset of 3511 vaginal births following spontaneous labour (Figure 1). As this project sought to evaluate outcomes following delivery in water, four cases of intra-uterine death prior to delivery (all subsequently delivered on land) were excluded. The final analysis included 3507 babies born alive.

Pre-specified neonatal outcomes included need for neonatal unit admission prior to discharge from hospital, low Apgar score (defined as less than 8 to identify even minor reductions in score) at 1 and 5 minutes of age, temperature within the first hour following delivery, and neonatal mortality.

Outcomes were compared for babies delivered in water and those not delivered in water to assess the safety of waterbirth. Apgar scores (dichotomised as not normally distributed) and neonatal unit admission were compared using a chi-squared test. Mean temperature after delivery was compared using a t-test. Analyses were conducted using Stata and Clinstat.

As this was a service evaluation, National Health Service Research Ethics Committee approval was not required, as determined by the Health Research Agency online decision tool [12].

## Results

In the period 1 April 2014 to 31 March 2016 there were 592 waterbirths and 2915 non-waterbirths (Table 1). There was no significant difference in maternal age or sex of baby. There was no difference in median gestation between the groups, although mean birthweight was 90 g higher in the waterbirths group. The majority of waterbirths occurred in the midwife-led birthing centre, whilst non-waterbirths were split more evenly between the midwife-led unit and main delivery suite. Mothers delivering in water were less likely to have had two or more previous pregnancies.

**Table 1:** Baseline statistics. a) p-value for chi-squared test for trend

Variable	Waterbirths	Non-waterbirths	All births	p-value
Maternal age (mean [standard deviation])	30.1 (5.1)	29.8 (5.3)	29.8 (5.3)	0.19
Gestation (median [inter-quartile range])	40 + 1 (39 + 4, 40 + 5)	40 + 1 (39 + 2, 40 + 5)	40 + 1 (39 + 2, 40 + 5)	
Place of birth				
Birthing suite	564 (95.2%)	1174 (40.3%)	1738 (49.6%)	
Delivery suite	11 (1.9%)	1648 (56.5%)	1659 (47.3%)	
Obstetric theatre	0	5 (0.2%)	5 (0.1%)	
Home	16 (2.7%)	69 (2.4%)	85 (2.4%)	
Other/unknown	1 (0.2%)	19 (0.7%)	20 (0.6%)	
Birthweight (kg; mean [standard deviation])	3.54 (0.42)	3.45 (0.45)	3.46 (0.45)	<0.0001
Sex of baby				
Female	287 (48.5%)	1434 (49.2%)	1721 (49.1%)	0.75
Male	305 (51.5%)	1480 (50.8%)	1785 (50.9%)	
Number of previous pregnancies				
0	167 (28.4%)	775 (26.8%)	942 (27.0%)	0.0003^a^
1	242 (41.0%)	1030 (35.6%)	1272 (36.5%)	
2	120 (20.4%)	558 (19.3%)	678 (19.5%)	
3+	60 (10.2%)	533 (18.4%)	593 (17.0%)	

There was no significant difference in rates of neonatal unit admission between those born in water and those not (Table 2). There is evidence of higher one-minute Apgar scores in the waterbirth group, although this attenuated by five minutes of age. Mean temperature following delivery did not differ between groups. Three infants in the waterbirths groups had temperatures less than 36 degrees Celsius (all 35.9), compared to 14 in the non-waterbirths group (range 32.5 to 35.9). Of the two patients admitted to the neonatal unit following waterbirth, one was discharged back to routine midwifery care after a brief period (less than four hours) of observation, and the other was treated for a respiratory diagnosis with no persisting morbidity beyond discharge. All babies in the waterbirth group were alive at discharge. In the non-waterbirth group, 31 babies were admitted to the neonatal unit: 18 with primarily respiratory symptoms, eight meeting criteria for possible hypoxic-ischemic encephalopathy, two for brief observation/senior paediatric review (less than four hours) before discharge back to midwifery care, one requiring transfer to a surgical centre. For two babies the reason for admission could not be identified from electronic records. One baby admitted with respiratory symptoms did not survive.

**Table 2:** Early neonatal outcomes. a) p-value for chi-squared test b) p-value for t-test

Variable	Waterbirths	Non-waterbirths	p-value
Neonatal unit admission (all)	2 (0.3%)	31 (1.1%)	0.10^a^
Admission with respiratory symptoms	1 (0.2%)	18 (0.6%)	
1-minute Apgar <8	27 (4.6%)	200 (6.9%)	0.04^a^
5-minute Apgar <8	4 (0.7%)	33 (1.1%)	0.32^a^
Temperature (mean; degrees Celsius)	36.7	36.8	0.23^b^

Twenty-one cases (0.6%; two waterbirths, 19 non-waterbirths) were missing data on 1-minute Apgar scores and 22 cases (0.6%; two waterbirths, 20 non-waterbirths) were missing 5-minute scores. 218 cases (6.2%) were missing temperature data; rates were similar for waterbirths (5.7%) and non-waterbirths (6.3%).

Of the 1572 cases excluded due to induced labour, three of these subsequently delivered in water; of the nine excluded due to missing data about the onset of labour, two of these subsequently delivered in water. These five infants born in water all had normal Apgar scores at one minute (range 8 to 9) and five minutes (range 9 to 10), a normal temperature within the first hour (range 36.5 to 37.1 degrees), and did not require neonatal unit admission.

## Discussion

An evaluation of neonatal outcomes in uncomplicated vaginal deliveries in a UK District General Hospital has demonstrated that giving birth in water had outcomes which are comparable to birth on land. It is important for maternity services to be able to demonstrate that services are safe, and also for staff to be able to provide accurate information on outcomes when helping women and their partners make decisions around delivery.

These findings are consistent with existing research studies which have suggested similar outcomes in those born in water compared to those on land [1,9,10]. A strength of this evaluation is the use of prospectively collected hospital data and the relatively large sample size obtained over a 24-month period. Use of temperature measurement and neonatal unit admission as outcomes provided objective outcome measures. One of the two babies admitted to the neonatal unit following waterbirth was treated for a respiratory diagnosis; however, respiratory conditions were the most common cause for admission across all births, and actual rates of respiratory admissions were higher among non-waterbirths. Overall neonatal admission rates were low, reflecting the relatively low-risk population being studied; to study causes for admissions in greater detail would require a far larger sample obtained across multiple centres.

Limitations of this work include potential bias through the selection of the comparison group of non-waterbirths – it is possible that this group includes more complex pregnancies and therefore has a higher risk for neonatal complications. This was minimised through limiting the dataset to unassisted vaginal deliveries following spontaneous onset of labour; however, the potential for some residual bias cannot be fully excluded. The majority of waterbirths occurred on the birthing suite, with the location of non-waterbirths spread more evenly. It is possible that there could be differences in the way that Apgar scores are recorded by staff in different locations [13], possibly introducing a recording bias. However, staff members are trained in Apgar scoring, and the Apgar outcomes are consistent with the other outcome measures.

Although the gestational age of infants did not differ between groups, mean birthweight was slightly higher among those born in water. However, this difference of 90 g is unlikely to be of clinical significance. Temperature measurements were missing for approximately 6% of cases. Although rates of missing data were similar between waterbirths and non-waterbirths, we cannot fully exclude potential bias introduced through missing data. Apgar scores were recorded for more than 99% of cases.

As this project sought to evaluate safety within a single hospital trust in the UK, it is unclear how generalisable these findings may be to other hospitals or healthcare settings. A multi-centre observational study comparing delivery in water to delivery on land would be helpful in this regard, as a randomised-controlled study is unlikely to be feasible or acceptable to labouring women.

## Conclusions

An evaluation of safety in a UK District General Hospital has shown that giving birth in water is comparable to giving birth on land in terms of Apgar score, temperature after delivery and rates of neonatal unit admission, for low-risk spontaneous vaginal delivery. Evaluation of longer-term outcomes, such as readmission to hospital following discharge from postnatal services, would be helpful to see if outcomes remain similar beyond the newborn period. Evaluation in different units would help guide how generalisable these findings may be to other hospitals or healthcare settings.
